# Double Tropopauses and the Tropical Belt Connected to ENSO

**DOI:** 10.1029/2020GL089027

**Published:** 2020-07-14

**Authors:** Hallgeir Wilhelmsen, Florian Ladstädter, Torsten Schmidt, Andrea K. Steiner

**Affiliations:** ^1^ Wegener Center for Climate and Global Change (WEGC) University of Graz Graz Austria; ^2^ FWF‐DK Climate Change, University of Graz Graz Austria; ^3^ Institute for Geophysics, Astrophysics, and Meteorology/Institute of Physics (IGAM/IP) Graz Austria; ^4^ GeoForschungsZentrum Potsdam (GFZ) Potsdam Germany

**Keywords:** UTLS, Double tropopause, ENSO, Satellite observations, Tropical belt, Subtropical jet stream

## Abstract

A detailed analysis of double tropopause (DT) occurrences requires vertically well resolved, accurate, and globally distributed information on the troposphere‐stratosphere transition zone. Here, we use radio occultation observations from 2001 to 2018 with such properties. We establish a connection between El Niño‐Southern Oscillation (ENSO) phases and the distribution of DTs by analyzing the global and seasonal DT characteristics. The seasonal distribution of DTs reveals several hotspot locations, such as near the subtropical jet stream and over high mountain ranges, where DTs occur particularly often. In this study, we detect a higher number of DTs during the cold La Niña state while warmer El Niño events result in lower DT rates, affecting the structure of the tropopause region. Close to the Niño 3 region, this relates to a much lower first lapse rate tropopause altitude during La Niña and corresponds to an apparent narrowing of the tropical belt there.

## Key Points

- Double tropopause characteristics and the connection to ENSO are analyzed using vertically high resolved GPS RO observations
- More double tropopauses are detected during the cold La Niña phase and less during El Niño
- During La Niña, a much lower first lapse rate tropopause corresponds to a narrowing of the tropical belt around the Niño 3 region

## 1 Introduction

The tropopause, the transition zone between the troposphere and the stratosphere, is of substantial importance to the exchange between these two atmospheric regimes (Holton et al., 1995). Depending on season and latitude, the tropopause is typically found at around 16 km in the tropics and at around 9 km at high latitudes (e.g., Schmidt et al., 2005; Seidel & Randel, 2006). At midlatitudes, the higher tropical tropopause domain may overlap the lower high‐latitude tropopause domain and form double tropopauses (DTs), either because the high‐latitude tropopause domain extends equatorward (Peevey et al., 2014; Wang & Polvani, 2011) or because the tropical tropopause domain extends poleward (Homeyer et al., 2010; Castanheira et al., 2012; Liu & Barnes, 2018; Pan et al., 2009; Randel et al., 2007). The more complex structure and variability of the upper troposphere and lower stratosphere (UTLS) region at midlatitudes, related to this overlap and the existence of DTs there, is key to understand the stratosphere‐troposphere exchange (e.g., Boothe & Homeyer, 2017).

DT events are found especially frequently at midlatitudes in both hemispheres, in storm track regions, on the poleward side of the subtropical jet stream (STJ), more frequently during winter (Bischoff et al., 2007; Schmidt et al., 2006; Seidel & Randel, 2006), and over land (Schmidt et al., 2006). The STJ is of special interest, not only because it marks a region where midlatitudinal and tropical air meet, but the STJs are linked to Rossby wave breaking events, which again are associated to DTs and stratosphere‐troposphere exchange (e.g., Castanheira & Gimeno, 2011; Peevey et al., 2012). The exchange is enhanced when DTs are present (Liu & Barnes, 2018).

DTs have also been found in association with mountain gravity waves (Schmidt et al., 2006), cyclogenesis (Añel et al., 2008) and the upward vertical motion in warm conveyor belts in strong cyclonic circulation systems (Peevey et al., 2014; Wang & Polvani, 2011). DTs are linked to the strength of the upward branch of the Brewer‐Dobson circulation (Castanheira et al., 2012) and are detected in cloud‐top inversion layers (Biondi et al., 2012).

Previous studies (e.g., Reid & Gage, 1985; Rieckh et al., 2014) have shown a strong correlation between the altitude of the first lapse rate tropopause (LRT) and the El Niño‐Southern Oscillation (ENSO). Castanheira et al. (2012) showed a clear signal of both the quasi‐biennial oscillation (QBO) and the ENSO in DT frequencies from reanalyzed data (ERA‐Interim).

However, a systematic analysis of globally distributed DTs from observations and their link to ENSO phases has not yet been conducted. Previous studies on multiple tropopauses have mainly used radiosonde or reanalysis data. Due to the sparse sampling of radiosondes, a global view on the characteristics of DTs is hardly possible. Data with lower vertical resolution or smoother temperature profiles tend to smear out DT features and underestimate DT frequencies (Biondi et al., 2012; Manney et al., 2017; Vergados et al., 2014; Xian et al., 2019). Since the early 2000s, however, globally distributed measurements from radio occultation (RO) are available. This limb sounding technique provides temperature profiles with high accuracy and vertical resolution in the UTLS for applications in atmospheric research and climate (Anthes, 2011; Steiner et al., 2011). Some studies have used a subset of available RO data to study DTs (e.g. Lakkis & Canziani, 2009; Randel et al., 2007; Schmidt et al., 2006; Xu et al., 2014).

In this study, we take advantage of the precise DT detection with RO to present the global and seasonal characteristics of DTs from observations. For the first time, we analyze the relation between DTs and ENSO events and its implication on the tropopause structure based on the recent full multiyear RO record, which now covers 11 ENSO events. This larger sample substantially improves our ability to investigate these relations.

## 2 Data

Due to their high vertical resolution and global distribution, RO satellite measurements are well suited for investigating the thermal tropopause. RO measurements from different missions can be combined and used in continuation of each other (Foelsche et al., 2011; Schreiner et al., 2007). We used the Wegener Center OPSv5.6 data set (Angerer et al., 2017), a compilation of most RO satellite missions to date, enabling us to study DTs over a longer time period than previous studies. In this study, we use temperature profiles from September 2001 to December 2018, interpolated to an evenly spaced, fixed vertical grid with *h*=100 m between the grid points.

El Niño and La Niña events are identified using the Oceanic Niño Index (ONI), which is a ±1 running mean of monthly mean sea surface temperature anomalies in the Niño 3.4 region (5°S to 5°N and 170°W to 120°W). An event is called “El Niño” or “La Niña” when five succeeding months of the ONI are all above 0.5 K or all below −0.5 K, respectively.

In addition, we indicate the location of the STJ using the maximum mean horizontal wind speed between 200 and 300 hPa within 5° latitude × 5° longitude grid cells from European Centre for Medium‐Range Weather Forecasts (ECMWF) 6‐hourly analysis wind fields.

## 3 Methods

The standard definition of the World Meteorological Organization (WMO, 1957) was used to calculate the thermal LRTs: “(a) The first tropopause is defined as the lowest level at which the lapse rate decreases to 2°C/km or less, provided also the average lapse rate between this level and all higher levels within 2 km does not exceed 2°C/km. (b) If above the first tropopause the average lapse rate between any level and all higher levels within 1 km exceeds 3°C/km, then a second tropopause is defined by the same criterion as under (a). This tropopause may be either within or above the 1 km layer.” This algorithm was applied to each RO temperature profile on an evenly spaced 100‐m grid. The search started from below, using a latitude, *φ*, dependent lower altitude limit, *z*_start_ (in meters), following

(1) $$z_{\text{start}}(\varphi )=\text{6,250}\;m+\text{1,250}\;m\times \cos\varphi .$$

This limit was adapted from Son et al. (2011) and adjusted downwards to include more LRTs at midlatitudes. The search was terminated at 25 km.

A candidate LRT altitude was first found on the evenly spaced 100‐m vertical grid, according to the thresholds in the WMO (1957) definition. Due to the implementation, this point was always found at an altitude above the threshold. To avoid a positive bias, the altitude was fine tuned, by linear interpolation, down to the altitude where the lapse rate equals Γ=2°C km^−1^. This altitude was selected to represent the LRT.

The number of first LRTs, *N*_1_, and the number of second LRTs, *N*_2_, from all the available temperature profiles were counted and the corresponding DT percentages calculated using

(2) $$\text{DT percentage}=\frac{N_{2}}{N_{1}}\times 100\%.$$

We calculated these percentages within 5° × 5° grid cells and within each 5° zonal band or each 5° meridional band. Finally, monthly mean DT anomalies were created relative to the mean seasonal cycle from 2007 to 2018.

## 4 Results

### 4.1 Global DT Occurrences

**Figure 1:**
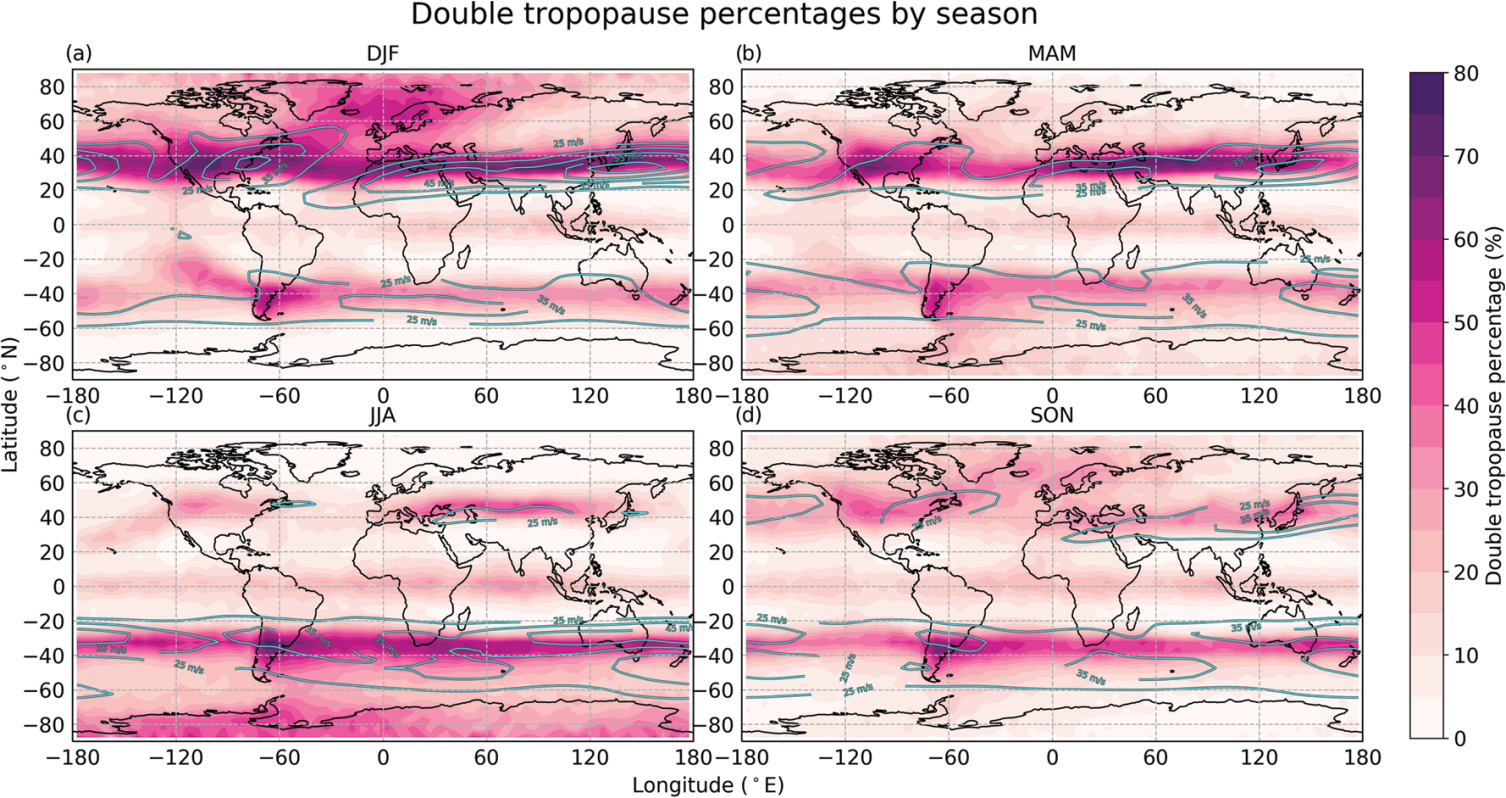
Global spatial distribution of DTs from the years 2001 to 2018 by season: (a) December‐January‐February (DJF), (b) March‐April‐May (MAM), (c) June‐July‐August (JJA), and (d) September‐October‐November (SON). Cyan contour lines: Mean winds (of the maximum between 300 and 200 hPa) stronger than 25 ms^−1^ are indicated, with 10 ms^−1^ between the contours.

The main characteristics of the global DT distribution can be deduced from Figure 1. The figure reaffirms previous studies (e.g., Peevey et al., 2012, 2014; Randel et al., 2007; Schmidt et al., 2006; Wang & Polvani, 2011; Xu et al., 2014) and shows that the features are consistently revealed in the multiyear RO record. The seasonal features have been described in greater detail in Peevey et al. (2012). DTs are mainly found at midlatitudes along the STJ and are more frequent during winter and over land. The strong belt of DTs in the STJ regions gets weaker during summer, on both hemispheres, due to the weaker eddy activity as the summer STJ slows down. The STJs are primarily radiatively driven and move equatorward during winter (e.g., Maher et al., 2020; Manney et al., 2014), which is also the case for the location of the DT belt during December‐January‐February (DJF) (Figure 1a) and June‐July‐August (JJA) (Figure 1c).

The enhanced DT percentages in the tropical regions for all seasons may be explained by cloud tops (Biondi et al., 2012) or gravity waves in the stratosphere (e.g., Hoffmann et al., 2013 and references therein), both caused by deep convection in the tropics. Additionally, some of the DTs detected in the tropics are related to changing QBO phases (e.g., Kedzierski et al., 2016). We also detect these QBO‐related DTs because we use a rather high upper altitude limit of 25 km when finding the LRTs, which is several kilometers above the mean LRT altitude, well into the QBO region in the stratosphere.

Furthermore, Figure 1 reveals locations where DTs are found particularly often. In the Northern Hemisphere (NH), such hotspots are located east of the Rocky Mountains, over the Himalayas, and over Japan. In the Southern Hemisphere (SH), they are found over and east of the southern Andes and over southeast Australia. All these DT hotspots are found on the STJ band, on the lee side of high mountains. They get weaker during summer, supporting the source to be mountain gravity waves related to the STJ (Schmidt et al., 2006). The enhancement east of Japan more or less disappears during JJA but is globally the strongest hotspot during DJF. The hotspot leeward of the Southern Andes is, as an exception, prominent for all seasons and is globally the strongest hotspot during JJA. The area is known for its high number of occurrences of gravity waves (Ern et al., 2018; Hoffmann et al., 2013; Sato et al., 2012).

To the *west* of the Andes, there is a DT *tail*, pointing toward the tropics, that only shows up during DJF (Figure 1a). As the main STJ flow is eastward, the tail is on the windward side of the Andes and therefore requires an alternative explanation. There may also be a similar, somewhat weaker, feature during JJA (Figure 1c) in the northeastern Pacific, west of the Rocky Mountains, also pointing toward the tropics.

### 4.2 Annual Patterns of DTs

**Figure 2:**
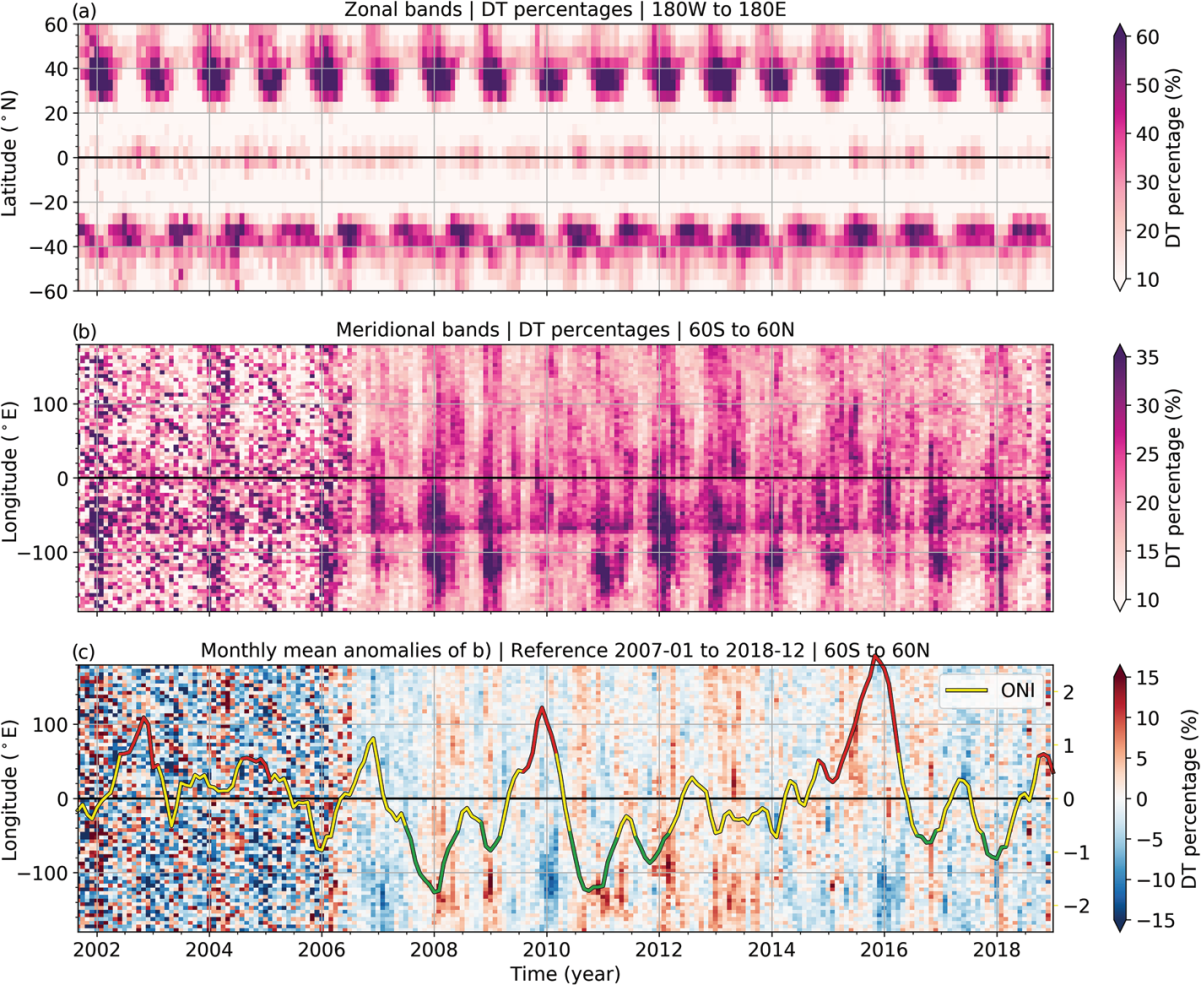
Monthly time series of DT percentages split into (a) 5° zonal bands, (b) 5° meridional bands, and (c) monthly mean anomalies of the meridional bands in panel (b). Only the DTs found between 60°S and 60°N were included in panels (b) and (c). Line in panel (c): The Oceanic Niño Index (ONI), with La Niña events in green and El Niño events in red. The right *y* axis in panel (c) corresponds to the ONI, in Kelvin.

The seasonal DT development is exposed in Figure 2 in zonal (Figure 2a) and meridional (Figure 2b) bands. The noisy structure before 2006 is caused by the lower number of RO measurements in the early RO phase (see, e.g., Steiner et al., 2011), especially apparent in the meridional view in Figure 2b. The seasonality and clear zonal DT distribution mainly in the subtropics of both hemispheres become evident in Figure 2a. In both hemispheres, an annual pattern of peaks in DT numbers moves equatorward during winter, stopping at around ±30° latitude and withdrawing poleward in summer. The DT rates in the NH are more pronounced, last longer, and cover a greater area than in the SH, due to the different land‐sea distribution and thus different Rossby wave patterns (e.g., Schmidt et al., 2006; Xian et al., 2019).

The prominent straight line in Figure 2b, around 75°W, is attributed to the hotspot leeward of the southern Andes that shows up in every season in Figure 1. In contrast, the dip just west of 75°W reveals a meridional band with rarely any DTs, just to the west of the Andes, from the beginning to the end of the time series.

The irregularly recurring patterns further west in Figure 2b, between 150°W and 90°W, resemble ENSO time patterns. The peaks mainly show up between 30°S and 10°S and again between 10°N and 30°N, which is just north and south of the Niño 3 region (5°N to 5°S, 150°W to 90°W). Both the locations and the pattern suggest a link to the ENSO. In the following, we unravel more details and possible explanations.

### 4.3 ENSO and DT Structure

The relation between ENSO and DT occurrences becomes evident in Figure 2c. First, it reveals that the warmer El Niño events result in lower DT rates. The indication of an ENSO connection is especially prominent for the El Niño events in 2009/2010 and 2015/2016. The ONI peak in 2006/2007 is not called an El Niño event because the values did not last long enough above the 0.5 K threshold, but nevertheless, the rate of DTs is also reduced. Second, the colder La Niña events lead to more DTs, especially between 150°W and 90°W.

**Figure 3:**
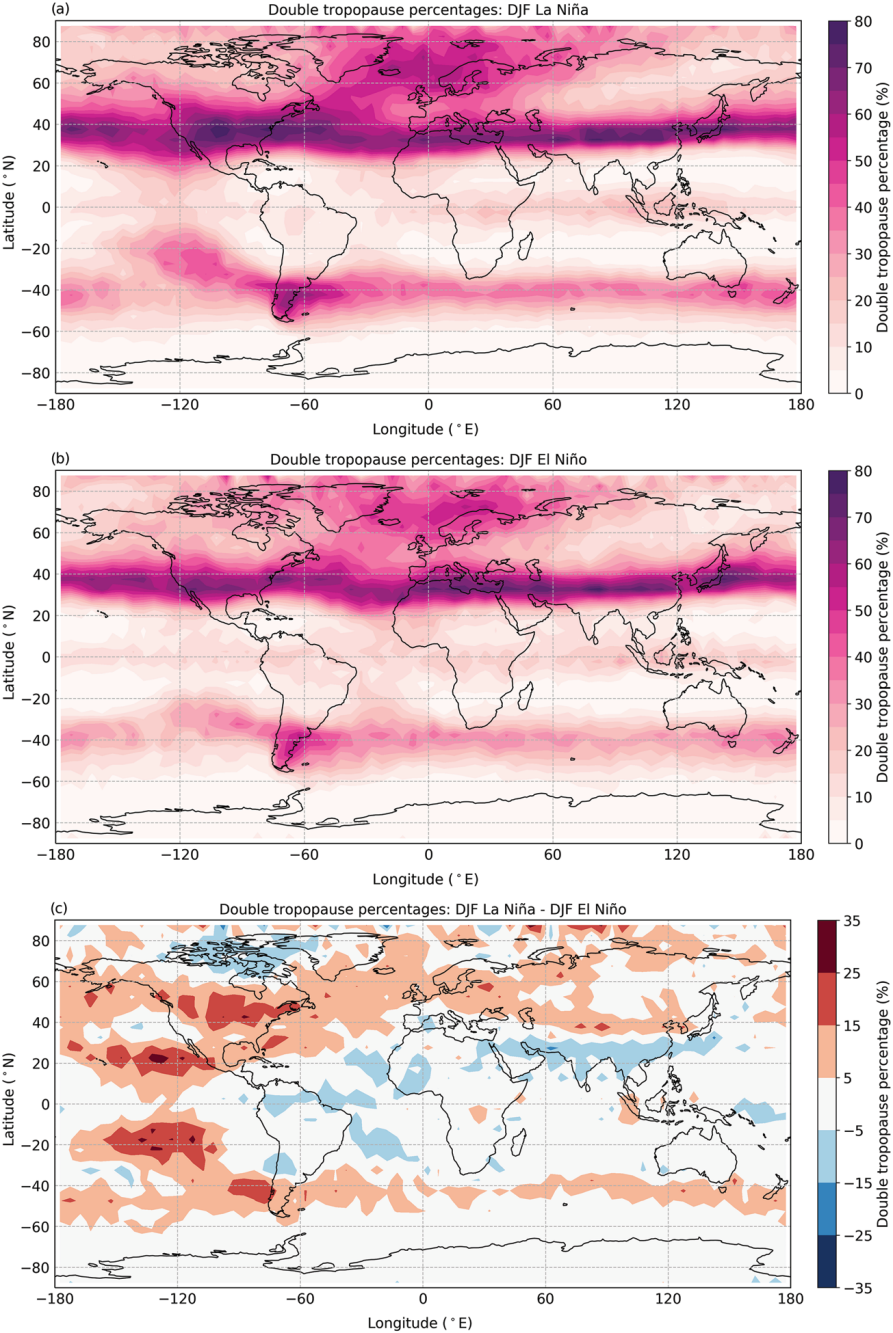
Global spatial distribution of DTs during (a) DJF La Niña, (b) DJF El Niño, and (c) difference between DJF La Niña and DJF El Niño (i.e., a minus b).

For further investigation of the DT and ENSO relation, we limit the considered DTs to the DJF seasons only and examine El Niño and La Niña periods separately. This minimizes the seasonal influence on the difference between the two ENSO states. ENSO events occur more frequently during DJF, with 16 La Niña and 16 El Niño months detected in the considered time period. Figure 3 shows the global spatial distribution of DTs split into DJF La Niña events (Figure 3a) and DJF El Niño events (Figure 3b). It appears that the DT tail to the west of the Andes (cf. Figure 1a) mainly originates from the DJF La Niña time periods, as it is much weaker during DJF El Niño and the other time periods (not shown). Figure 3c shows the difference between the DT percentages during DJF La Niña and DJF El Niño, that is, the difference between Figures 3a and 3b. It uncovers that the tail is not only a SH feature but also shows up on the NH, disguised (in Figure 3a) by the high frequency of DTs in the latitudinal band around the NH STJ during DJF.

The increased DT occurrences in the eastern Pacific region during La Niña compared to El Niño periods are caused by distinct atmospheric circulation regimes. During La Niña, the upwelling part of the Walker circulation is located over the Maritime continent, while during El Niño the main upwelling moves to the central Pacific (see, e.g., Gettelman et al., 2001; Lau & Yang, 2015). An analysis of atmospheric parameters related to cyclonic activity (divergence and vorticity; not shown here) confirmed that during La Niña (El Niño), cyclonic (anticyclonic) activity is dominant in the tropopause region above the eastern Pacific. According to Randel et al. (2007), upper tropospheric level cyclonic vorticity is related to an enhancement of DT occurrence and lower LRT heights compared to anticyclonic vorticity at the same altitude level. This is in good agreement with the observed differences in Figure 3c.

Figure 3 additionally suggests that during DJF La Niña, more DTs should be expected at locations where they are usually found than during DJF El Niño. The regions just west of the Andes hotspot and east of the Rocky Mountains hotspot especially stand out. The enhancement east of Japan, on the other hand, seems to be unaffected by ENSO events. There is a southward shift around the Himalayan hotspot during DJF El Niño, also seen between the seasons (Figure 1), related to jet stream shifts (Maher et al., 2020).

### 4.4 Narrowing the Tropical Belt During La Niña

**Figure 4:**
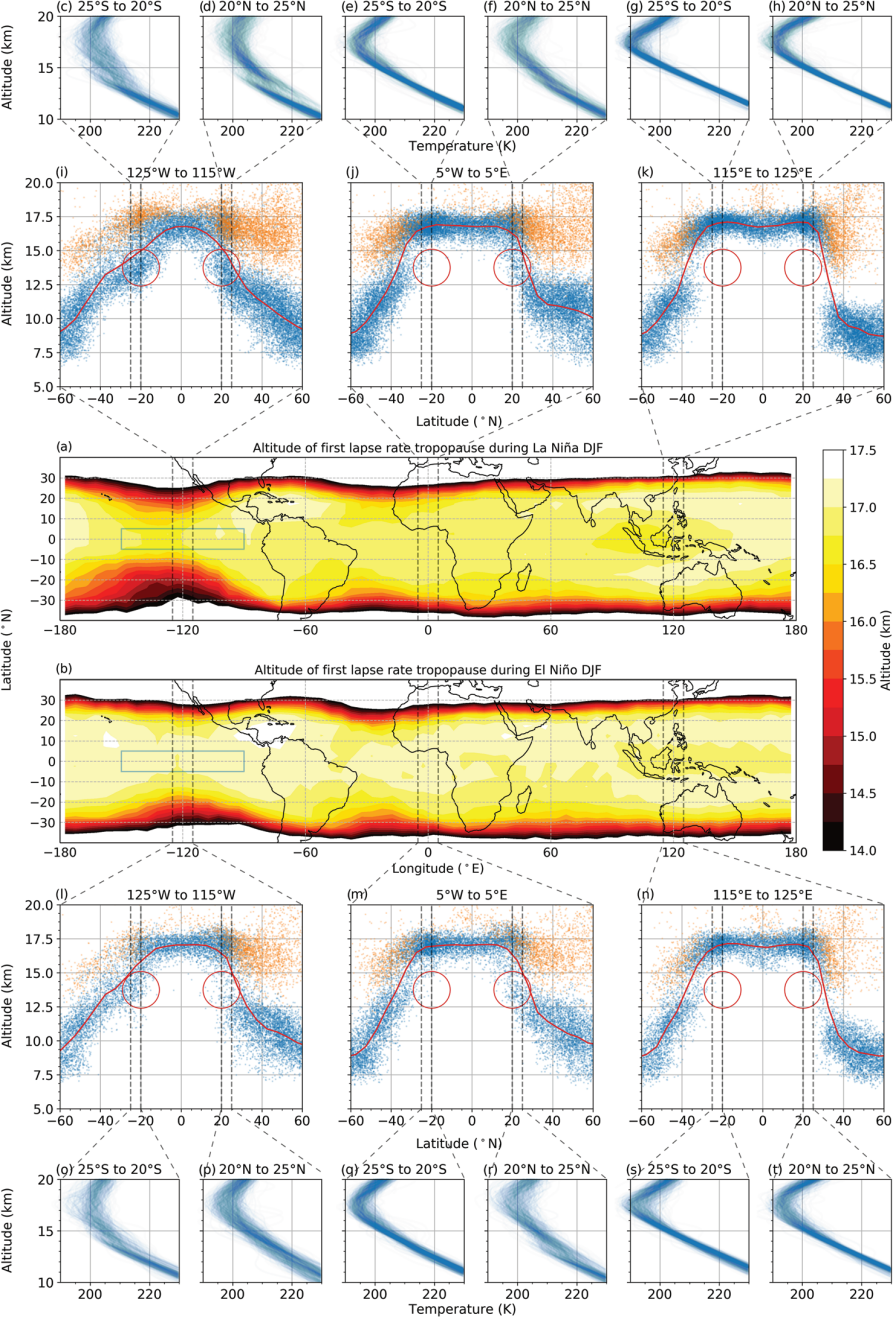
Middle panels (a and b): The mean altitude of the first LRT for DJF only, during (a) La Niña and (b) El Niño. The blue rectangles mark the location of the Niño 3 region. Second topmost (i, j, k) and second bottommost panels (l, m, n): The location of all the first (blue) and the second (orange) LRTs found within the meridional bands marked by dashed lines in the middle panels. Red lines: The mean altitudes of the first LRT within these dashed lines. The red circles have the same location in each panel, for comparison. Topmost (c to h) and bottommost (o to t) panels: The temperature profiles from 25°S to 20°S and 20°N to 25°N (i.e., the profiles within the dashed lines in panels i to k and l to n).

We investigate the impact of ENSO conditions on the tropopause structure in Figure 4. Figures 4a and 4b show the mean of all the *first* LRT altitudes during DJF La Niña and DJF El Niño, respectively. For most longitudes, the mean first LRTs in the tropics are found at a slightly lower altitude during DJF La Niña (Figure 4a) than during DJF El Niño (Figure 4b). This is in agreement with Randel et al. (2007) (see above). Poleward of the Niño 3 region, they are exceptionally low, corresponding to a remarkable narrowing of the tropical belt around those longitudes. Figure 4i shows the altitude of all the first (blue) and second (orange) LRTs within a 10° meridional band at the Niño 3 region (leftmost dashed meridional band in Figure 4a), at their corresponding latitudes during La Niña. Additional tropopauses appear below the tropical tropopause domain (approximately  20°S to 20°N) from what seems to be an equatorward expansion of the high‐latitude tropopause domain. The tropical LRT domain is still present, as a mix of first and second LRTs, but the mean first LRT appears much lower than usual in the specified region for DJF La Niña. The corresponding temperature profile cluster plots in Figures 4c–4h (DJF La Niña) and Figures 4o–4t (DJF El Niño) support these observations. This becomes especially clear in Figure 4d, where the temperature profiles start off with a steady lapse rate until they are sliced in two “branches” at the first LRT and break into typical, although less sharply defined, tropical temperature profiles (cf. Figure 4g). For comparison, the regular tropopause characteristics are depicted for two additional meridional bands in Figure 4.

Similar to the DT frequency as described in the previous section, the altitude of the first LRT is also influenced by the circulation patterns. As cyclonic vorticity leads to lower first LRT altitudes (Randel et al., 2007), the shifting cyclonic circulation patterns during La Niña, induced by the change in the Walker circulation, are therefore considered to be connected to the observed tropical belt narrowing.

## 5 Conclusions

To summarize, high vertical resolution and global coverage make RO satellite measurements well suited for studying DTs. The measurements are especially accurate in the altitude range of the tropopause. We exploited these characteristics to investigate for the first time the relation of ENSO and the occurrence of DTs in global multiyear RO observations.

We demonstrated that findings from previous studies are consistently revealed and presented a seasonally and regionally resolved picture of various DT hotspots. DTs are mainly triggered along the STJ in winter, especially prominent over the Rocky Mountains, the Himalayas, and the Andes.

Temporal, resolved DT patterns introduced a connection between ENSO and DT occurrences. It revealed an increase in DTs to the west of the Andes, poleward of the Niño 3 region (5°N to 5°S, 150°W to 90°W). We found that the strength and location of this increase is evidently connected to the ENSO. Colder La Niña events lead to a higher number of DTs while warmer El Niño events result in lower DT rates. This difference in DT occurrences is considered to be caused by the changing atmospheric circulation regimes of the Walker circulation between the ENSO phases.

During La Niña, the higher number of DTs detected at the Niño 3 region seems to be an equatorward expansion of the high‐latitude tropopause domain. This leads to a mix of first and second LRTs at the edge of the tropical belt and a mean first LRT altitude that is much lower than usual. This corresponds to an apparent narrowing of the tropical belt there.

Knowledge of the detailed structure of the UTLS region is of great relevance for the analysis of the stratosphere‐troposphere exchange. Identifying regions of increased DT occurrences points to a possible enhanced exchange. This has implications for the composition of the atmosphere, influencing, for example, the radiative balance and the dynamics of the atmosphere (see, e.g., Stohl et al., 2003 and references therein). The enhanced DT frequencies and lower first LRTs suggest that these processes are of increased relevance in the tropical Eastern Pacific during La Niña.

Recent studies have discussed the widening of the tropical belt (e.g., Staten et al., 2018 and references therein). This widening is difficult to determine due to large internal variability. The dependence of the tropical belt width on ENSO presented in this study might be of relevance to future studies on this topic.

Compared to neutral ENSO phases, ENSO events substantially alter the UTLS structure at midlatitudes and the tropics. For a detailed analysis, vertically high resolved information with global coverage is needed. Our results show that RO observations are able to provide such analysis and contribute to gaining improved knowledge of the transition between troposphere and stratosphere and the variability of the tropical belt.
